# Editorial: Functionally-adapted biomaterials for diagnosis, treatment and prevention

**DOI:** 10.3389/fbioe.2023.1234330

**Published:** 2023-08-14

**Authors:** Weiguang Yin, Yao Xiao, Shen Liu

**Affiliations:** Department of Orthopedic Surgery, Shanghai Sixth People’s Hospital Affiliated to Shanghai Jiao Tong University School of Medicine, Shanghai, China

**Keywords:** biomaterial, electrospinning, hydrogels, exosomes, silk fibroin membrane

Biomaterial science studies biological materials and their interactions with the biological environment. Therefore, Biological material as a kind of adjustability material can be used to diagnose and treat the diseases of an organism. The Research Topic *Functionally-adapted Biomaterials for Diagnosis, Treatment and Prevention* aims to illustrate the wide application of functionally-adapted biomaterials in regenerative medicine, drug delivery, prevention, diagnosis, treatment, and many other fields.

Multifunctional polymer biomaterials are widely used in biomedical fields, among which is known for its simplicity, efficiency and flexibility. Electrospinning can be used to manufacture nanofibers and nanomaterials with a variety of topologies. Ge et al. demonstrated that combining electrospinning and self-emulsification could improve the dissolution of low water-soluble anticancer model drugs (paclitaxel, PTX). By analysing scanning electron microscopy and transmission electron microscopy (TEM) results, they found that Core-shell nanofibers fabricated by coaxial blending process could provide a better self-emulsifying process with a higher encapsulation efficiency and a better drug sustained release profile. In addition, increasing the thickness of the sheath section also positively affected the self-emulsifying properties. Finally, this paper provides a new thought for oral administration of poorly water-soluble drugs and demonstrates the superiority of functionally-adapted biomaterials used as a drug carrier.

Hydrogels have been applied in tissue regeneration research due to their excellent biocompatibility and plasticity. Hu et al. had reported a review of applications of functionally-adapted hydrogels in tendon repair. In this review, they firstly presented their knowledge on the mechanisms of tendon healing. Then they enumerated the obstacles encountered in applying hydrogels in tendon repair (such as poor mechanical properties and side effects of degradation). In addition, they also discussed the issues related to regulating the differentiation of tendon stem cells and polarization of macrophages by changing the physicochemical properties of the hydrogel.

In addition to hydrogels, exosomes have also been studied in tissue repair and drug delivery. Various researches have shown that exosomes play a role in angiogenesis, tissue repair and reconstruction. Pan et al. described the feasibility of applying hydrogels and exosomes from material source, classification, and performance in bone tissue repair. More importantly, they also comprehensively analyzed the application prospect of nanohydrogels coated exosomes in tendon healing. By complementing each other’s advantages, hydrogels coated exosomes are expected to play a greater role in diagnosing and treating orthopedic diseases.

Silk fibroin membrane can be used in medical diagnostics due to its high porosity and large specific surface area. Li et al. fabricated a simple, rapid, reliable, sensitive, and cost-effective method for prenatal detection of fetomaternal haemorrhage by combining multi-aperture silk membrane with enzyme-linked immunosorbent assay (ELISA). The presence of anti-A and anti-B fetal red blood cells in maternal peripheral blood was determined by visual color of material. This research shows that functionally-adapted biomaterials can rapidly diagnose diseases in clinical practice and take measures without delay.

All four papers have shown the efforts of investigators of many fields to advance biomaterials. With the deepening understanding on disease regulation mechanisms and the continuous innovation of biomaterials, functionally-adapted materials will have a broader prospect in future clinical treatment.

